# Gluteal Propeller Perforator Flaps: A Paradigm Shift in Abdominoperineal Amputation Reconstruction

**DOI:** 10.3390/jcm12124014

**Published:** 2023-06-13

**Authors:** Theodoros Chrelias, Yanis Berkane, Etienne Rousson, Korkut Uygun, Bernard Meunier, Alex Kartheuser, Eric Watier, Jérôme Duisit, Nicolas Bertheuil

**Affiliations:** 1 Department of Plastic, Reconstructive and Aesthetic Surgery, South Hospital, CHU Rennes, University of Rennes 1, 35700 Rennes, France; tchrelias@gmail.com (T.C.); yanis.berkane@chu-rennes.fr (Y.B.); etienne.rousson@chu-rennes.fr (E.R.); eric.watier@chu-rennes.fr (E.W.); jerome.duisit@gmail.com (J.D.); 2 Vascularized Composite Allotransplantation Laboratory, Massachusetts General Hospital, Shriners Children’s Boston, Harvard Medical School, Boston, MA 02115, USA; korkut.uygun@gmail.com; 3 Center for Engineering in Medicine and Surgery, Massachusetts General Hospital, Harvard Medical School, Boston, MA 02115, USA; 4 MICMAC, UMR INSERM U1236, Rennes University Hospital, 35033 Rennes, France; 5 Department of Hepatobiliary and Digestive Surgery, CHU Rennes, University of Rennes 1, 35700 Rennes, France; bnielss@aol.com; 6 Colorectal Surgery Unit, Department of Surgery, Cliniques Universitaires Saint-Luc, 1200 Brussels, Belgium; alex.kartheuser@uclouvain.be; 7 Department of Plastic and Reconstructive Surgery, Hôpitaux IRIS Sud, 1050 Brussels, Belgium

**Keywords:** abdominoperineal amputation, perineal reconstruction, pelvis, perforator flaps, propeller flaps

## Abstract

Abdominoperineal amputation (AAP) is a gold standard procedure treating advanced abdominal and pelvic cancers. The defect resulting from this extensive surgery must be reconstructed to avoid complications, such as infection, dehiscence, delayed healing, or even death. Several approaches can be chosen depending on the patient. Muscle-based reconstructions are a reliable solution but are responsible for additional morbidity for these fragile patients. We present and discuss our experience in AAP reconstruction using gluteal-artery-based propeller perforator flaps (G-PPF) in a case series. Between January 2017 and March 2021, 20 patients received G-PPF reconstruction in two centers. Either superior gluteal artery (SGAP)- or inferior artery (IGAP)-based perforator flaps were performed depending on the best configuration. Preoperative, intraoperative, and postoperative data were collected. A total of 23 G-PPF were performed—12 SGAP and 11 IGAP flaps. Final defect coverage was achieved in 100% of cases. Eleven patients experienced at least one complication (55%), amongst whom six patients (30%) had delayed healing, and three patients (15%) had at least one flap complication. One patient underwent a new surgery at 4 months for a perineal abscess under the flap, and three patients died from disease recurrence. Gluteal-artery-based propeller perforator flaps are an effective and modern surgical procedure for AAP reconstruction. Their mechanic properties, in addition to their low morbidity, make them an optimal technique for this purpose; however, technical skills are needed, and closer surveillance with patient compliance is critical to ensure success. G-PPF should be widely used in specialized centers and considered a modern alternative to muscle-based reconstructions.

## 1. Introduction

Abdominoperineal amputation (APA) is currently widely performed to treat locally advanced cancers (i.e., rectal, urinary system, gynecological, cutaneous) [1,2,3], endometriosis [4], major infections (such as Fournier gangrene [5], Hidradenitis Suppurativa [6]), inflammatory bowels diseases [7] and chronic fistulas [8,9]. Perineal defects after APA have always been a challenge for surgeons due to their particular location. Indeed, primary closure is often difficult, and the management of bowel exposure complicates controlled wound healing. The technical approach is based on various criteria, such as morbidity, estimated procedure duration, and provisional rehabilitation. After primary closure, perineal wound complications, such as wound infection, dehiscence, or delayed healing, will occur in up to 67% of the cases [10,11]. Moreover, when radiation therapy is performed, the perineal morbidity increases [12]. The first reconstructive techniques relied on muscular and musculocutaneous flaps. Among them, the vertical rectus abdominis myocutaneous (VRAM) flap [13,14,15] was the most employed. Nowadays, VRAM is still considered a gold standard and a workhorse flap for its fast and easy-going harvesting, especially when a laparotomy is required for the digestive procedure. In the new era of robotic and minimal-invasive abdominal surgery for APA, however, techniques sparing the abdominal wall should be promoted. Propeller perforator flaps based on gluteal vessels likely represent the best option and the first choice to replace VRAM. Propellers, such as any perforator flaps, which are widely used for the last two decades, help reduce morbidity by only harvesting sub-cutaneous tissues. In our present study, we investigated the possibility of reconstructing moderate to extensive APA with gluteal-artery-based propeller perforator flaps (G-PPF).

## 2. Materials and Methods

### 2.1. Patients and Extracted Data

We report our experience in 20 patients with 23 G-PPFs after an APA, between January 2017 and March 2021 in the Plastic and Reconstructive Surgery department of Rennes University Hospital (Rennes, France) and the General Surgery department of Saint-Luc University Hospital UCLouvain (Brussels, Belgium).

The APA procedure was performed by senior colorectal surgeons, and reconstruction with G-PPF by two senior plastic surgeon authors (NB, JD). All patients were classified by The American Society of Anesthesiologists (ASA) physical status classification system. Two types of perforator flaps were performed: the superior gluteal artery perforator (SGAP) and the inferior gluteal artery perforator (IGAP) flaps. For each patient, we recorded the following data: age, sex, body mass index (BMI), smoking status, history of the loss of substance, and comorbidities. Recorded data involved the following: procedure duration, loss of substance dimensions, flap dimensions, and number and type of perforator arteries. Reported complications leading to surgical revision or special postoperative management were divided into two groups:

- Flap-related complications, such as arterial suffering, venous congestion, partial or total flap necrosis, hematoma, wound dehiscence, infection, or fat necrosis;
- Systemic complications, such as deep venous thrombosis, pulmonary embolism, need for transfusion, neoplasia recurrence, and death.

### 2.2. Surgical Techniques

The intra-abdominal approach following APA was performed by laparotomy (*n* = 9, 45%), laparoscopy (*n* = 5, 25%), and robot-assisted surgery (*n* = 6, 30%). Previous publications described patient-based indications for APA approaches [16]. Regarding the choice of performing the excision surgery with or without robot-assisted laparoscopy in our study, the decision could be decided based on the tumor characteristics (size, lymph node status, location), the patient’s nutritional status and overall general condition, and the surgeon’s fluency with the robot.

In our series, the perineal reconstruction was achieved either primarily (*n* = 8, 40%) during the APA or secondarily (*n* = 12, 60%) to treat delayed wound dehiscence after primary direct perineal closure. Perforator flap harvesting was only performed last, after both abdominal and perineal steps, since the patient often had to be turned in a prone position to provide optimal surgical access for SGAP harvesting. The IGAP flap was preferred for fragile patients because it could be procured in a supine position. Classically, SGAP perforators are detected on the two median thirds on a line drawn from the posterior superior iliac spine to the greater trochanter [17], and IGAP perforators are detected above the inferior gluteal fold near the ischium. It is important to note that for propeller perforator flaps techniques, random perforators were searched nearby the wound defect with an 8 MHz acoustic handheld Doppler (Hadeco ES 100 VX, Farla, Belgium, E.U) (Video S1). To harvest an SGAP flap, perforator vessel detection was performed at the posterior part of the defect, whereas to harvest an IGAP flap, the perforator vessel detection was performed close to the anterior part of the perineal defect to allow reconstruction with a propeller flap rotated 90°. A compression test was performed to increase Doppler-detection specificity [18,19]. The preoperative elliptical flap drawing was based on the location of these perforators as a pivot point for the rotation. The length of the flap corresponds to the distance between the pivot point and the distality of the loss of substance to be covered. The minimum width of the flap was taken as ¼ of the length, which has to be adequate to fill the defect’s width and ideally obtain a direct closure of the donor site. A first incision of up to 3 cm was performed to dissect and isolate the perforators. If the vessels were not found and/or their caliber was too small, the dissection was pursued either on the other gluteal side or on the other group of perforators (superior gluteal to inferior or inferior to superior). The perforating pedicle was isolated with a vessel loop, and the flap was elevated subfascial until reaching the isolated perforators (Video S2). Once the flap was totally elevated, a rotation test was performed. If the range of motion was insufficient and/or too much tension was observed on the pedicle, the perforator dissection was pursued intra-muscularly until optimal flap mobility and a limited pedicle tension were obtained. Required rotation was 90° up to 120° in our series, following the Tokyo consensus [20]. The flap skin edges were sutured with loose half-buried horizontal mattress sutures (3/0 Ethilon, Ethicon, Inc., Raritan, NJ, USA) in order to anticipate the flap edema formation within the first 24–72 h; indeed, tight sutures can be responsible for propeller flap suffering [21]. The donor site was closed primarily in three planes with interrupted absorbable sutures (Polysorb 2; COVIDien; and Monocryl 3/0; Ethicon Inc.) and a running suture (Monocryl 4/0) over suction drains. Postoperatively, the patients were hospitalized in the department of plastic surgery to be monitored hourly for the first 2 days, then three times a day until the end of the first week. Monitoring focused on the aspect of the flap’s skin (color, heat, capillary refill time, skin flexibility). To avoid any pedicle extrinsic compression, patients were kept in prone and alternate lateral decubitus positions. In every case, the dressing of both the flap and the donor site was changed once a day. The drains were removed until the drainage was reduced to a small amount (30 mL or less for two days in a row). All patients received low molecular weight heparin (LMWH) to prevent venous thromboembolism 15 days postoperatively. The mobilization of the patient could be started after the first 48 h.

### 2.3. Data Collection and Statistics

All patient-related data were transferred to GraphPad Prism 9 (La Jolla, CA, USA), which was used for all statistical analyses. Descriptive statistics resulted from the dedicated function of the software. The quantitative data are presented with mean, standard deviation, and ranges, when relevant.

### 2.4. Ethics Approval Statement

The study was performed in compliance with the principles of the Declaration of Helsinki (1964) and the French bioethics laws that have been applied since 7 July 2011. This study did not require a specific approval from our local ethics committee. 

## 3. Results

**Figure 1:**
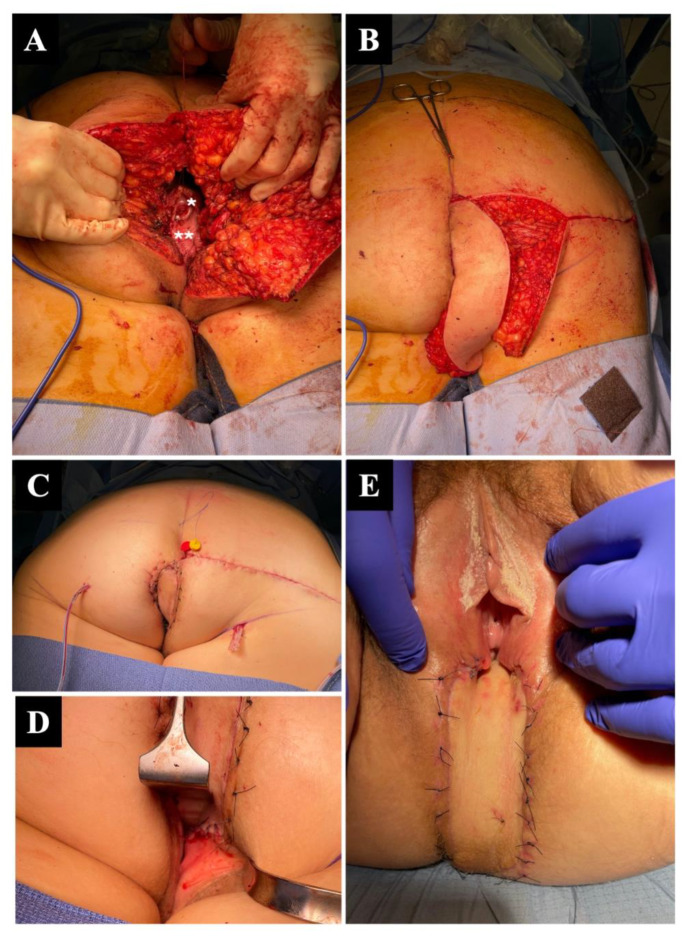
Perineal reconstruction following abdominoperineal amputation (AAP) for rectal adenocarcinoma, involving the complete vaginal posterior wall, in a 42-year-old woman. The amputation was performed by a combined robotic laparoscopic and open perineal approach (**A**). The patient was then placed in a prone position for reconstruction, with visible remaining cervix (*) and remaining anterior vaginal wall (**). (**B**) A 23 × 6 cm inferior gluteal artery perforator IGAP flap was harvested and rotated by 90°. (**C**) Completed reconstruction, with flap in position and direct closure of the gluteal donor area. (**D**,**E**) Immediate and 1-week aspect of the reconstructed perineum and posterior vaginal wall with the IGAP flap.

**Figure 2:**
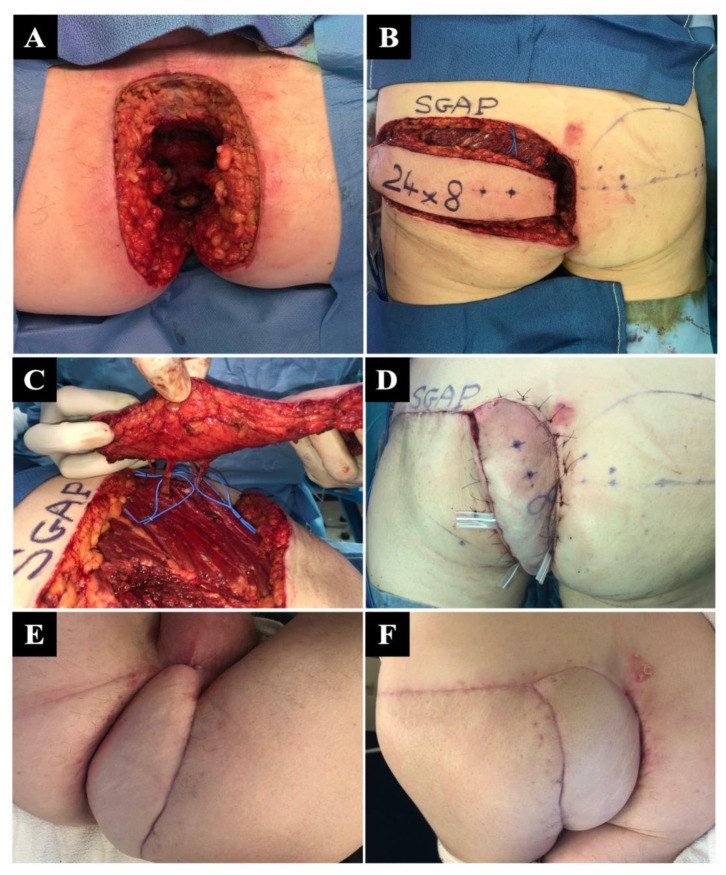
Major perineal reconstruction for epidermoid carcinoma of the anal canal in a 64-year-old man. (**A**) Massive defect after a combined robotic and direct perineal amputation. (**B**) Procurement of a single 24 × 8 cm superior gluteal artery perforator SGAP flap in a prone position before rotation. (**C**) A 90° rotation of the flap based on three large superior gluteal perforating vessels. (**D**) Immediate aspect after flap inset and gluteal donor site closure, with mild venous congestion. (**E**,**F**) A 2-month follow-up showing complete healing and defect closure.

**Figure 3:**
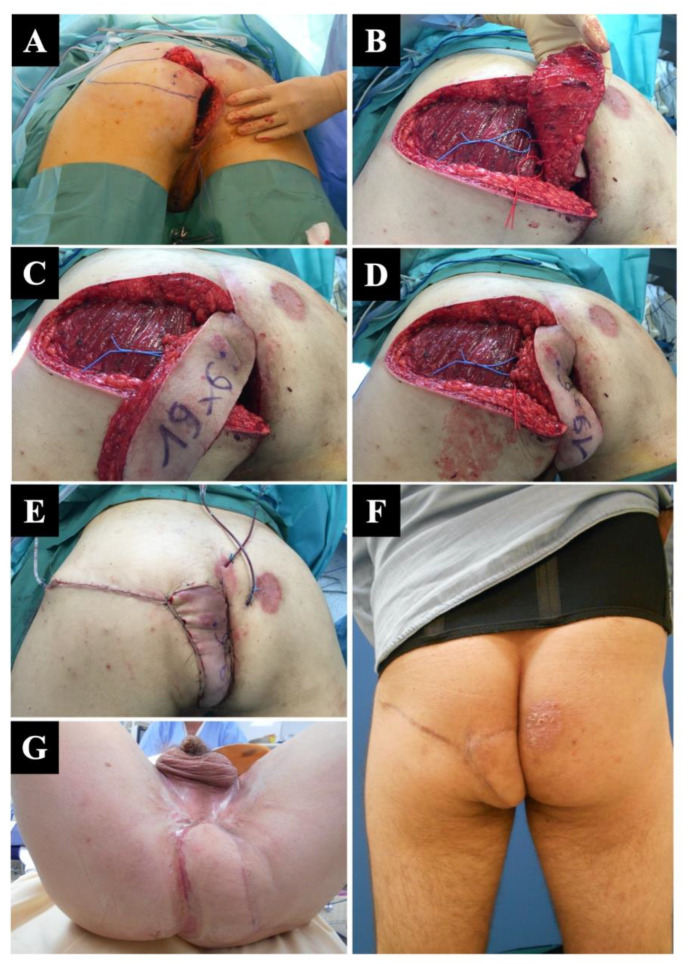
Deep perineal defect following abdominoperineal amputation AAP in a 43-year-old man with an epidermoid carcinoma of the anal canal. (**A**) Final appearance of the perineum following AAP. (**B**) The medial border of the defect was used to detect two perforator vessels from the superior gluteal artery. (**C**) A full-thickness superior gluteal artery perforator SGAP flap was harvested and rotated with a 90° angle to (**D**) cover the defect. (**E**) Immediate aspect of the flap was satisfactory. (**F**) Follow-up control after 6 weeks confirming the success of the reconstruction. (**G**) Full healing after 4 weeks, as seen in the supine view.

**Figure 4:**
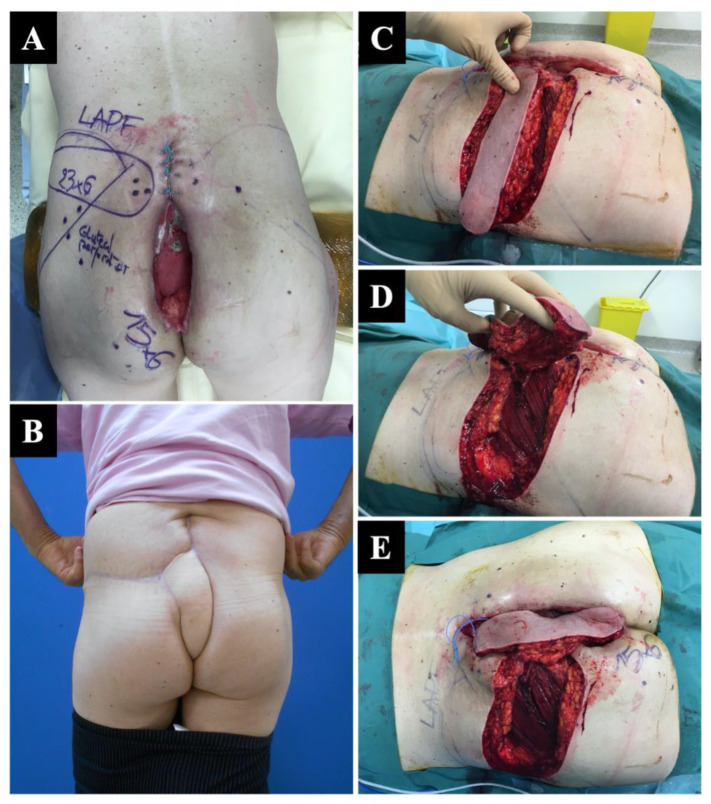
Delayed (secondary) reconstruction after major scar dehiscence following abdominoperineal amputation AAP in a 61-year-old man. (**A**) An extensive preoperative perforator mapping was performed, and a lumbar artery perforator flap (LAPF) flap was initially planned; however, the LAPF perforator vessels were not found intraoperatively, and the choice was made to choose a superior gluteal artery perforator (SGAP) flap as the most suitable solution. (**B**) SGAP flap harvesting. (**C**) Minor intramuscular dissection allowing a 90° rotation (**D**). (**E**) The patient fully healed, achieving a good functional and cosmetic outcome.

In our series, 20 patients received reconstruction following abdominoperineal amputations. The mean age of included patients was 62.6 ± 10.8 (range 42 to 82 years) years old. The mean BMI was 23.9 ± 3.5 kg/m^2^ (range 18 to 29.8). None of the patients smoked at the time of reconstructive surgery. ASA scores were 3 (*n* = 8), 2 (*n* = 9), and 1 (*n* = 3) (Table 1). The indications were rectal adenocarcinoma (*n* = 9), anus epidermoid carcinoma (*n* = 7), and other cancer (*n* = 4). A total of 23 G-PPF were performed—12 SGAP and 11 IGAP flaps (illustrative Figure 1, Figure 2, Figure 3 and Figure 4). In one case, two flaps had to be harvested concomitantly to allow closure. For two patients, a second flap was harvested secondarily to achieve reconstruction (one flap failure due to arterial injury of the pedicle per operatively, another flap failure due to postoperative venous congestion, which led to total flap necrosis). The average loss of substance was 15.6 × 6.3 cm (range 35 to 192 cm^2^). The mean flap skin paddle was 17.4 cm × 3.8 cm (range 60 to 192 cm^2^). The average surgery duration was 232 ± 74 min (range 128–360 min). Donor sites were closed directly in every case.

Eleven patients experienced at least one complication (55%). The defect coverage success rate was 100%, with three patients (15%) experiencing at least one flap complication; one perioperative arterial vessel injury and two venous congestion treated by leech therapy [22] evolved into partial distal necrosis of the flap (treated by secondary healing) and one total flap necrosis (which led us to harvest a second perforator flap). Six patients had delayed healing, one patient underwent a new surgery at 4 months for a perineal abscess under the flap, and three patients died from disease progression.

## 4. Discussion

For a long time, it was believed that muscular tissue was necessary to fill the pelvic cavity after an APA, leading to the frequent use of muscular flaps. As mentioned before, the rectus abdominis muscle used to be the gold standard in this type of reconstruction [13,14,15]. Other muscular flaps have been described, such as the gracilis [11,23], gluteus muscles [24], and even latissimus dorsi as a free flap [25]; however, the perineal defect following surgical resection does not always require a large flap, and the muscle tissue does not necessarily bring more filling to the emptied pelvic space. Moreover, any transposed and denervated muscle will present a subsequent atrophic evolution. With the G-PPF approach, the pelvic cavity behind the fasciocutaneous flap is filled by the descent of the intestine. For superficial perineal defects where deep defect filling is unnecessary, one possible approach for reconstruction involves utilizing acellular dermal matrices and split-thickness skin grafts [26]. This method is particularly applicable when treating hidradenitis suppurativa [27], a condition that can result in sufficiently large defects but infrequently involves complete removal of perineal tissue. Even in such cases where the contouring using skin grafts is not satisfactory [28], the utilization of perforator flaps remains a dependable and repeatable technique for reconstruction [27,29,30].

In our series, all APA defects were covered with propeller SGAP and IGAP flaps, which therefore confirms them as a valuable and robust first option for both wound closure and cavity filling. In our study, all the SGAP flaps were raised with two to three perforators, and all the IGAP flaps were raised with one or two perforators. These flaps are well described in the literature, mainly as free perforator flaps for breast reconstruction [31,32,33,34,35]. The anatomical study by Ahmadzadeh R. et al. showed several perforating vessels [36], while three perforator groups were identified in SGAP flaps described by Guerra [34]. When the patient is prone, the available perforators emerge from the superior gluteal artery (SGAP) or the inferior gluteal artery (IGAP) [37]. In supine position, another type of perforator flap can be used for perineal reconstruction based on the internal pudendal artery perforator (IPAP) vessels [38]. The Singapore flap (neurovascular pudendal thigh flap) is useful but may not supply sufficient volume after extended pelvic exenteration [39]. In the case of a laparotomy-based approach, the pedicled deep inferior epigastric artery perforator (DIEP) flap is emphasized as a first-choice flap for APA reconstruction [40,41]. If the defect is more important, the reconstruction can combine perforator flaps, either using a similar contralateral flap or two different flaps [42,43,44,45]. Even then, G-PPFs provide a better aesthetic outcome compared to double V-Y fasciocutaneous advancement flap approaches [46].

Intra-operative flexibility is critical, and postdebridement Doppler evaluation is important to determine if the vascularization of the planned flap is confirmed. As we showed in our series (Figure 4), this intraoperative assessment can lead to a change in the approach. This adaptation is critical but also simple since all the gluteal-based perforator flaps can be harvested in the same position.

Another reason to prefer propeller perforator flaps is that they are an attractive option in the era of abdominal wall-sparing approaches after general surgery procedures. gluteal propeller flaps seem to be the best combination with laparotomy or laparoscopic/robot-assisted resections. Reconstructive surgeons can therefore avoid harvesting rectus abdominal-based flaps as a first choice. In addition, in the case of a concomitant laparotomy, muscle-sparing solutions with pedicled deep inferior epigastric perforator (DIEP) flaps should be advocated [40,41]. Εven if Agochukwu et al. [47] described the laparoscopic harvesting of the rectus abdominis muscle, avoiding a long skin incision, the morbidity related to muscle harvesting still remains, as well as hernias and parietal weaknesses described in up to 82% of the cases [48].

We observed a 55% complication rate in our series, but only one major complication led to a surgical revision. These results are in favor of the low morbidity of this muscle-sparing approach. Indeed, one of the major advantages of these flaps is the reduction of donor-site morbidity. Despite their recent consideration as a primary method for reconstructive surgery by plastic surgeons, the complication rates are low, as demonstrated with large clinical studies involving propellers perforator flaps [42,49]. An innovative and interesting approach to further improve outcomes and prevent complications, such as minor wound dehiscence or partial flap necrosis, could involve the injection of platelet-rich plasma into the flap borders. This technique has been described by some authors and shows potential [50,51]. Other approaches, such as ischemic preconditioning, have been studied, but the lack of clinical studies makes it a scarce approach that needs further evidence [52,53,54,55]. One crucial consideration is the intraoperative mapping of the flaps, which can be accomplished using various technologies, such as indocyanine green (ICG) angiography [56,57,58] or evaluation through microvascular Doppler ultrasound [59].

However, the learning curve for perforator-based flap harvesting is steep, and performing a musculocutaneous flap remains the fastest and safest way for the surgeon to reconstruct the pelvis. Perforator-based approaches should indeed be performed in specialized centers for APA treatments by expert plastic surgery teams. Moreover, some cancer patients are very fragile and often non-eligible for long-lasting anesthesia (as needed for the meticulous perforator dissection) associated with extended prone positions. Revision surgeries can then also be decreased due to the lower technicity needed. In these complex patients, where the comorbidities linked to the flap’s donor site are of secondary importance, an abdominal muscle flap could remain the safest choice, especially in non-expert centers; however, our results support that the quality of the reconstruction should, on the other hand, never be a criterion to favor muscle flaps over perforator flaps.

One limitation of this procedure is that postoperative pain related to sciatic nerve exposure can occur, especially after procuring IGAP flaps [36]. This was also described by Guerra et al. [34]; however, we did not observe any nerve injury in our patients, as the deep intramuscular dissection of the vessels remained superficial. Our results show that the minor complications resulting from G-PPF procedures are mostly due to vein issues. The surgeon needs to proceed with the vein selection very carefully, and the venous caliber may be more important than the arterial one, influencing the flap harvesting pattern. The propeller rotation indeed provokes vein compression more than arterial compression due to the thin venous vessel wall. As we know from Salgarello et al., the risk of thrombosis in a microanastomosis can be increased more than 10% in the artery and more than 200% in the venous in a 270 deg twisting [18]. Chaput et al., in a series of 228 patients undergoing propeller flaps, minimized the complication rate due to venous congestion to only 2.19% by allowing a 120° rotation of the flap by skeletonizing the vessels when it was necessary [60]. Koulaxouzidis et al. adopted another approach by strictly avoiding extensive skeletonizing of the pedicle to decrease risk of venous congestion. They showed only minor would healing disorders as complications in about 23.6% of their patients. In our series, where most of the flap underwent 180° rotation, the venous issue was solved by pursuing minor intramuscular dissection of the pedicles if needed perioperatively. A critical step was the postoperative management of the patient and their positioning in the bed in order to avoid compression of the pedicle [61]. In addition, leech therapy was used if needed [21], eventually allowing full recovery of the flap’s physiology.

Reconstructive surgeons face significant challenges when dealing with major perineal defects, primarily due to their location, the patient’s medical history, and the nature of transfixing resections that expose the intraabdominal cavity. In recent times, anterolateral thigh perforator artery (APA) flaps have emerged as a viable option in salvaging oncological recurrences following radiotherapy; however, in cases where APA wound dehiscence occurs, reconstructive procedures using abdominal flaps necessitate a laparotomy to transfer the flap into the defect. This procedure becomes even more delicate and risky if a prior laparotomy has been performed or if the patient is medically fragile. Furthermore, with the advent of laparoscopic and robotic approaches in the modern era, patients requiring secondary coverage typically possess an intact abdominal wall. Thus, they require comprehensive management of both the abdominal and digestive aspects. To avoid the need for a laparotomy, it is essential to re-evaluate the conventional paradigm of the abdominal-wall-based reconstruction as a secondary option. Additionally, as radiation therapy has become the standard adjuvant treatment, APA is now more frequently employed as a secondary strategy for managing recurrences. In other words, gastrointestinal surgeons increasingly rely on plastic surgeons to perform flap reconstruction for secondary wound dehiscence or as a preventive measure for high-risk patients. These developments have consequently led to a rise in the frequency of abdominal-wall-sparing flap procedures. We recommend using abdominal-based flaps such as DIEP and rectus abdominis muscle flap (VRAM or Taylor’s flap), only in case of open laparotomy, or if the gluteal perforator flaps are compromised. Alternatively, in fragile patients with poor general condition, surgeons may have to consider using muscle-based flaps, such as VRAM or Taylor’s flap or even gracilis flaps; however, when dealing with APA defects that can be addressed by either SGAP (superior gluteal artery perforator) or IGAP (inferior gluteal artery perforator) propeller flaps, we recommend opting for the SGAP flap. Our recommendation is based on the following factors:

- Firstly, the superior gluteal arteries tend to have more robust perforator vessels compared to the IGAP vessels. This is evident in their larger diameters, with a mean diameter of 3.38 mm (ranging from 2–4.5 mm) for superior gluteal artery perforators, as opposed to 1.44 mm (ranging from 0.6–2.5 mm) for perforators from the inferior gluteal artery [34,62].
- The larger diameter of the superior gluteal artery perforators results in a larger angiosome, which in turn allows for a larger skin paddle that can be vascularized by a single perforator. Consequently, the safety and likelihood of flap survivability are higher with the SGAP flap compared to the IGAP flap due to the larger vascular supply.

Propeller flaps are the most modern and refined technique described for pedicled flap reconstruction [20]. These strategies offer great flexibility and plasticity for defect closure, as well as a direct closure of the donor site without any muscular sequelae. It can be easily and quickly harvested and offer a certain versatility in surgical strategies, as the selection of several perforator vessels can be done closer to the defect. Moreover, the dissection can stay superficial, and a deep intramuscular perforator dissection is not often mandatory. G-PPFs can help achieve robust reconstructions with well-vascularized tissue and can address large defects, as described in the literature [42,63] as well as our series. These flaps can be used not only for extensive perineal defect reconstructions but also for vaginal posterior wall replacement after partial or total loss [41,45,64]. We hope our results help break the dogma stating that a muscle is absolutely needed for defect filling. The goal of the reconstruction is to provide a well-vascularized tissue, as well as an easily conformable entity to reconstruct an anatomic appearance. It is possible that in the future, more advanced acellular dermal matrixes [65] or 3D- and 4D-printed tissue engineering chambers [66,67] could be developed to effectively fill the cavity, serving as an additional valuable tool; however, perforator flaps, which provide both the necessary volume and skin quality for coverage, appear to be a durable and reliable solution. More studies may be needed to help draw an algorithm for perioperative selection of the flap type, taking into account the skin paddle of a specific venous territory [68]; however, we believe that the results presented in this study should prompt plastic surgeons involved in these complex reconstructions to opt for propeller perforator flaps. Our experience adds to recent data in the literature encouraging modern reconstructions that minimize morbidity while improving long-term functional and cosmetic outcomes.

## 5. Conclusions

This study allows us sharing our experience in covering perineal defects with two types of local pedicled perforator flaps based on the gluteal artery. Following the trends in plastic surgery, which tend to minimize donor site morbidity and avoid muscle harvesting, we believe the SGAP and IGAP flaps provide great results. We propose using these robust and reliable flaps as a challenging alternative to abdominal flaps and to keep using muscular flaps for rare cases of major complications or highly complex patients.

## Figures and Tables

**Table 1:** General patient data.

Patient	Flap	Gender	Age	BMI	Etiology	ASA	Abdominal Digestive Approach (Laparotomy, Laparoscopy, Robotic)	Loss of Substance Characterization	Primary or Secondary Reconstructive Procedure
1	1	Male	61	21.40	Rectal Adenocarcinoma	3	Laparotomy	AAP, sacrectomy	Secondary
2	2	Male	57	18.00	Rectal Adenocarcinoma	3	Laparotomy	AAP, total vaginal exclusion	Secondary
3	3	Female	57	22.10	Anus Epidermoid Carcinoma	3	Laparotomy	AAP	Secondary
	4	Female	57	22.10	Anus Epidermoid Carcinoma	3	Laparotomy	Immediate primary flap failure	Secondary
4	5	Male	54	18.40	Anus Epidermoid Carcinoma	3	Laparotomy	AAP	Secondary
5	6	Male	64	26.10	Rectal Adenocarcinoma	3	Laparoscopy	AAP	Secondary
6	7	Female	53	19.90	Cervical Uterus Adenocarcinoma	3	Laparotomy	AAP, pelvectomy	Secondary
7	8	Male	43	28.10	Anus Epidermoid Carcinoma	2	Laparoscopy	AAP	Primary
8	9	Female	79	22.30	Endometrium Adenocarcinoma	2	Laparotomy	AAP	Primary
	10							AAP, flap necrosis	
9	11	Male	57	23.40	Anus Epidermoid Carcinoma	3	Laparoscopy	AAP	Secondary
10	12	Male	82	25.90	Rectal Adenocarcinoma	3	Laparotomy	AAP, sacrectomy	Secondary
11	13	Male	69	27.90	Rectal Adenocarcinoma	2	Laparotomy	AAP, digestive fistula	Secondary
12	14	Male	71	29.80	Anus Epidermoid Carcinoma	2	Laparoscopy	AAP	Primary
13	15	Female	61	21.10	Anus Epidermoid Carcinoma	1	Laparoscopy	AAP	Primary
14	16	Male	70	21.70	Anus Adenocarcinoma	2	Robotic	AAP	Primary
15	17	Female	42	28.90	Rectal Adenocarcinoma	1	Robotic	AAP, total vaginal posterior wall	Primary
16	18	Male			Rectal Adenocarcinoma	2	Robotic	AAP	Secondary
17	19	Female	68	23.9	Rectal Adenocarcinoma	2	Robotic	AAP, total vaginal posterior wall	Primary
	20	Female	68	23.9	Rectal Adenocarcinoma	2	Robotic	Immediate primary flap failure	Primary
18	21	Male	57	27.70	Mucinous Adenocarcinoma	2	Robotic	AAP	Secondary
19	22	Male	64	25.20	Anus Epidermoid Carcinoma	2	Robotic	AAP	Primary
20	23	Male	64	23.20	Rectal Adenocarcinoma	1	Laparotomy	AAP	Secondary

BMI: Body mass index; ASA: American Society of Anesthesiologists score; AAP: Abdominoperineal Amputation.

## Data Availability

Data supporting the reported results can be found by contacting the corresponding author.

## Supplementary Materials

The following supporting information can be downloaded at: https://www.mdpi.com/article/10.3390/jcm12124014/s1, Video S1: SGAP perforator detection. Video S2: SGAP flap harvesting.
